# The utility of radiographs prior to pin removal after operative treatment of supracondylar humerus fractures in children

**DOI:** 10.1007/s11832-015-0673-9

**Published:** 2015-07-30

**Authors:** John A. Schlechter, Michael Dempewolf

**Affiliations:** Children’s Hospital of Orange County, 1201 W. La Veta Avenue, Orange, CA 92868 USA; Adult and Pediatric Orthopaedic Specialists, 1310 W. Stewart Drive, Suite 508, Orange, CA 92868 USA

**Keywords:** Supracondylar humerus fracture, Radiographs, Percutaneous pinning, Postoperative management

## Abstract

**Purpose:**

To determine the safety, utility, and efficacy of pin removal prior to radiographs during the postoperative care of surgically treated displaced pediatric supracondylar humerus fractures.

**Methods:**

Retrospective review of 532 children with supracondylar humerus fractures treated with closed reduction and pinning from 2007 to 2012. Group 1: children who had their splint/cast removed and radiographs prior to pin removal. Group 2: children with radiographs taken after removal. Data recorded and analyzed included: demographic and radiographic data at the time of surgery and at final follow-up, including the number of radiographs taken prior to pin removal and if pins were ever retained after radiographs were performed on the date of intended removal.

**Results:**

There was no difference between the groups’ demographics. The number of postoperative radiographs taken prior to pin removal ranged from zero to two. No statistically significant change in Baumann’s (*p* = 0.79) or lateral humeral capitellar angles (*p* = 0.19) was noted between the groups. No children in group 1 (0/438) had their pins retained after radiographs were taken on the date of intended removal.

**Conclusion:**

Obtaining postoperative radiographs prior to pin removal, although commonplace, is not necessary. If fracture stability is noted intraoperatively, and there is an uneventful postoperative course, it is safe and effective to discontinue immobilization and remove pins prior to X-ray. This is safely done without change in alignment or clinical sequelae. Doing so can aid in clinic flow, may decrease child anxiety, and limit multiple cast room visits.

**Level of evidence:**

Level III therapeutic study.

## Introduction

Supracondylar humerus fractures are widely considered the most common fracture of the elbow in children. The annual incidence has been estimated at 177.3 per 100,000 [1]. Closed reduction and percutaneous pinning is the standard treatment for displaced pediatric supracondylar humerus fractures. These pins are most often left outside of the skin to ease and hasten later removal in the office. Pin removal performed in the office setting has been demonstrated to be safe and acceptable, with most children experiencing little or no pain [2, 3].

Cast room procedures, including cast or splint removal, and pin removal can, however, cause significant anxiety in many children. High levels of anxiety can lead to complications related to the procedure and unpleasant office visits [4, 5]. To our knowledge, the safety and utility of pin removal prior to obtaining radiographs in the setting of postoperative management of supracondylar humerus fractures in children has not been studied. The purpose of this study was to determine the utility and necessity of obtaining radiographs prior to the removal of pins placed during the operative treatment of pediatric supracondylar humerus fractures.

## Materials and methods

Our Institutional Review Board approved the protocol for this project. No outside funding was received for this work. We retrospectively reviewed the medical records and radiographs of 934 children who underwent closed reduction and percutaneous pinning for type II and III supracondylar humerus fractures at our institution between January 2007 and December 2012. Fractures were classified using the Wilkins modification to the Gartland classification [6]. The Wilkins modification subclassifies type III fractures into those that are displaced posteromedially or posterolaterally [7].

Inclusion criteria for analysis were: patients with postoperative follow-up in our orthopaedic outpatient setting, adequate anteroposterior and lateral radiographs at time of surgery, and at date of final radiographic follow-up. Patients with incomplete follow-up, inadequate radiographs, and ipsilateral upper extremity fractures were excluded. A number of cases were excluded secondary to radiographs that were deemed inadequate, often due to projectional differences between intraoperative and postoperative radiographs. It was felt that these projectional differences, although slight, could lead to measurement errors, and, thus, these patients were excluded from review. A total of 532 children were, thus, included for review. These patients were divided into two groups for comparison. Group 1 consisted of children who had their splint/cast removed and radiographs taken prior to pin removal on the date of intended implant removal. Group 2 consisted of children whose radiographs were taken after their splint/cast and pins had been removed on the date of intended removal.

All surgeries were performed by one of six pediatric orthopaedic surgeons. Each surgery was performed via closed reduction with placement of two or three percutaneous smooth pins. There were no cases in which the implants were buried beneath the skin and all pins were left protruding through the skin for later removal in the outpatient setting. All pins were cut, bent, appropriately dressed, and covered with external immobilization. Pin number and size was determined based on attending preference with reference to patient size, fracture severity, and fracture stability. The anteroposterior and lateral fluoroscopic images were saved to the hospital’s picture archiving communication system (PACS). Patients were then immobilized in either a long arm splint or long arm fiberglass cast, based again on attending preference. The long arm fiberglass casts were either univalved or bivalved utilizing a cast saw. Patients were followed longitudinally through the outpatient clinic.

The number of radiographs taken prior to pin removal was recorded. Based on the operative surgeon’s custom and practice, radiographs were taken either prior to or after smooth pin removal, and this forms the basis of our study.

Radiographs taken during surgery and at the time of final radiographic follow-up were evaluated. Baumann’s and lateral humeral capitellar angles were measured, recorded, and compared. A single observer performed all radiographic measurements using digital angle software provided by Synapse, PACS (Fujifilm, Valhalla, NY, USA). Also recorded were the number of postoperative radiographs taken prior to pin removal, if pins were retained after radiographs were taken on the date intended for pin removal, and the number of postoperative days at which pins were removed.

Data analysis was performed to evaluate differences between the two groups. Demographic differences were determined using the Chi-square test. An analysis of variance (ANOVA) was used to compare the changes in Baumann’s and lateral humeral capitellar angles between the two groups. Statistical significance was determined using a *p*-value of 0.05.

## Results

Our final analysis included 532 operatively treated pediatric supracondylar humerus fractures in 532 children (241 girls and 291 boys), with a mean age of 4.9 years (range 2–13). There were 438 children in group 1, which consisted of children who had their splint/cast removed and radiographs taken prior to pin removal on the date of intended implant removal. Group 2 consisted of 94 children whose radiographs were taken after their splint/cast and pins had been removed on the date of intended removal.

The left extremity was involved in 59.7 % of the patients (318/532). A total of 216 (40.6 %) fractures were classified as type II and 306 (57.5 %) were type III. Extension type fractures comprised 522 (98.1 %) of all fractures, with only 10 (1.9 %) being flexion type. No demographic differences were noted between the two groups with regards to age, sex, extremity involved, and fracture type.

Fiberglass casts were used to immobilize 44.1 % of the patients (193/438) in group 1 and 98.9 % of the patients (93/94) in group 2 (*p* ≤ 0.001). Pins were removed in clinic at a mean of 26.1 days (range 11–49) postoperatively for group 1 and 27.2 days (range 18–42) postoperatively for group 2. Although there was a large range, this difference was not statistically significant (*p* = 0.96). Final radiographic follow-up occurred, on average, at 59.7 days (range 14–435) postoperatively in group 1 and 70.4 days (range 19–390) postoperatively in group 2.

No statistically significant change in Baumann’s or lateral humeral capitellar angles was noted between the two groups. The average measured change in Baumann’s angle from time of surgery to final radiographic follow-up was 2.33° in group 1 and 2.35° in group 2 (*p* = 0.799). The average changes in humeral capitellar angle between group 1 and group 2 were 2.58° and 2.59°, respectively. Again, this did not reach statistical significance (*p* = 0.199).

The number of postoperative radiographs taken prior to pin removal ranged from zero to two. This variability was dependent on attending preference. No change was noted in postoperative management in any of the 438 patients in group 1, irrespective of how many radiographs were taken. Notably, of the 438 children in group 1, none had their pins retained after radiographs were taken on the date of intended pin removal. No patients required a return trip to the operating room, and no patients were placed back into immobilization after pins were removed, with all children transitioning to a sling.

## Discussion

Supracondylar humerus fractures are widely considered the most common fracture of the elbow in children. Closed reduction and percutaneous pinning has become the standard of care for displaced fractures [1]. Pin removal is commonly and safely performed without anesthesia in the outpatient setting, with minimal pain [3].

There is variation in surgeon preference in the postoperative management of supracondylar humerus fractures. It has been accepted that a 3–4-week course of immobilization provides for adequate clinical and radiographic healing, and that the pins can be removed at that point. The average time to pin removal in our study was 27 days.

The timing and necessity of close radiographic follow-up is contentious. Ponce et al. retrospectively reviewed 104 patients and demonstrated no difference between those who had follow-up within 10 days and those who had delayed radiographic follow-up [8]. Karamitopoulos et al. demonstrated that, while mild alignment changes and pin migration could occur in the postoperative period, they have little effect on clinical management parameters or long-term sequelae [9].

There was a variation in postoperative follow-up observed in our cohort. Final radiographic follow-up occurred, on average, at 59.7 days (range 14–435) postoperatively in group 1 and 70.4 days (range 19–390) postoperatively in group 2. Although the reason for this was not specifically recorded for each individual, the wide range of follow-up may be attributed to several factors. At the discretion of the treating surgeon, some patients were followed longitudinally with scheduled visits past the 6- to 12-month postoperative mark. Some children were lost to follow-up soon after cast removal, resulting in a shorter follow-up time, and some children in the cohort presented for a new injury or care-giver concern and, therefore, longer term follow-up was able to be recorded.

Maximizing the quality of each radiograph performed on children and justifying the utility of the study is of paramount importance, especially when considering the desire to limit radiation exposure whenever possible. Children can become fearful and display signs of anxiety when visualizing exposed pins following elbow fracture surgical treatment. To lessen this, obtaining in-cast radiographs may lessen a child’s anxiety while undergoing postoperative radiographs following the operative treatment of supracondylar humerus fracture when compared to having radiographs obtained with exposed pins. Although fiberglass casts may allow for visibility and determination of alignment and healing in our experience when reviewing radiographs of children with elbow fractures following pin fixation, in-cast studies typically do not allow for “excellent” visualization and are often are of limited diagnostic quality due to the position of the arm while immobilized, as well as the overlying cast material obscuring the osseous structures and fracture healing. Therefore, if one wishes to obtain the highest quality postoperative imaging and best clarity, it is best done without the muddying of overlying cast material.

None of the 438 children who had their splint/cast removed and radiographs taken prior to pin removal on the date of intended implant removal had their pins retained after radiographs were taken. Therefore, we question the utility of this practice and now favor a protocol in which the removal of immobilization is followed by immediate implant removal and then out-of-cast radiographs on the date of intended pin removal, which, on average in this cohort, was 27 days.

Our study is not without limitations. Due to its retrospective nature, we could not report on patient-derived outcomes measures. As a retrospective review, there is a lack of standardization in the protocols for postoperative care. This lead to the variability in the type of immobilization, number of postoperative visits prior to pin removal, and number of postoperative radiographs, although this variability provided the basis of our study. One attending surgeon’s preference was to utilize fiberglass casts for immobilization and to remove pins prior to obtaining postoperative radiographs. This accounts for the statistical difference between the two groups with regards to the use of fiberglass casts. This, however, was not clinically significant, as there was no difference in the change of Baumann’s or lateral humeral capitellar angles between groups.

A single observer was used to review the 532 charts and perform the radiographic measurements. Overall, there was a small variation in the change of the Baumann’s and lateral humeral capitellar angles measured on radiographs taken during surgery and at the time of final radiographic follow-up. The small variance in measured change may be attributed to the large sample size of our cohort. Each measurement was performed three times and an average of the three measurements was taken and recorded as the final measurement. It has been previously demonstrated that utilizing a single observer for measurement of the Baumann’s and lateral humeral capitellar angles is a reliable and reproducible method for data collection. Silva et al. demonstrated excellent intraobserver reliability for the measurement of the Baumann’s angle (*r* = 0.8, *p* = 0.0001) [10].

To our knowledge, this is the first study to examine the utility of smooth pin removal prior to radiographic examination on the date of intended implant removal in children who have undergone operative treatment of a supracondylar humerus fracture. We present, through a retrospective review of a large cohort of 532 surgically treated pediatric supracondylar humerus fractures, minimal change in the Baumann’s and lateral humeral capitellar angles from the time of surgery to the final radiographic follow-up. No difference was observed in those patients who obtained radiographs after pin removal compared to those who obtained radiographs and then had their pins removed.

## Conclusion

Obtaining postoperative radiographs prior to pin removal, although routine, is not necessary. If fracture stability is noted intraoperatively and there is an uneventful postoperative course, it is safe and efficacious to remove the child’s postoperative splint or cast followed by immediate smooth pin removal prior to obtaining radiographs. We have demonstrated that this can be safely done with no significant change in radiographic alignment or clinical sequelae. Doing so can also aid in clinic flow and may decrease the child’s anxiety associated with obtaining radiographs with exposed pins and multiple cast room procedures.

## References

[1] Mulpuri K, Wilkins K (2012). The treatment of displaced supracondylar humerus fractures: evidence-based guideline. J Pediatr Orthop.

[2] Sorenson SM, Hennrikus W (2014). Pain during office removal of K-wires from the elbow in children. J Pediatr Orthop.

[3] Symons S, Persad R, Paterson M (2005). The removal of percutaneous Kirschner wires used in the stabilisation of fractures in children. Acta Orthop Belg.

[4] Katz K, Fogelman R, Attias J (2001). Anxiety reaction in children during removal of their plaster cast with a saw. J Bone Joint Surg Br.

[5] Liu RW, Mehta P, Fortuna S (2007). A randomized prospective study of music therapy for reducing anxiety during cast room procedures. J Pediatr Orthop.

[6] Gartland JJ (1959). Management of supracondylar fractures of the humerus in children. Surg Gynecol Obstet.

[7] Wilkins K, Rockwood C, King R (1984). Fractures and dislocations of the elbow region. Fractures in children.

[8] Ponce BA, Hedequist DJ, Zurakowski D (2004). Complications and timing of follow-up after closed reduction and percutaneous pinning of supracondylar humerus fractures: follow-up after percutaneous pinning of supracondylar humerus fractures. J Pediatr Orthop.

[9] Karamitopoulos MS, Dean E, Littleton A (2012). Postoperative radiographs after pinning of supracondylar humerus fractures: are they necessary?. J Pediatr Orthop.

[10] Silva M, Pandarinath R, Farng E (2010). Inter- and intra-observer reliability of the Baumann angle of the humerus in children with supracondylar humeral fractures. Int Orthop.

